# Examining Characteristics of Hospitalizations in Heart Failure Patients: Results from the 2009 All-payer Data

**DOI:** 10.23937/2469-5793/1510037

**Published:** 2016-06-28

**Authors:** Lufei Young, Carol Gilbert, Jungyoon Kim, Yaewon Seo, Fernando A Wilson, Li-Wu Chen

**Affiliations:** 1 College of Nursing, Augusta University, USA; 2 Department of Pediatrics, University of Nebraska Medical Center, USA; 3 Department of Health Service Research and Administration, University of Nebraska Medical Center, USA

**Keywords:** Heart failure, Readmissions, All-payer data, Rural health

## Abstract

Heart failure (HF) is one of the most common chronic and disabling illnesses, resulting in high morbidity and mortality. Readmission rate, one key indicator of healthcare quality and healthcare utilization, is prevalent in HF patients. Inconsistent evidences exist about the impact of rural health disparities on HF patients’ readmissions. The purpose of this explorative study was to examine the characteristics of hospitalized HF patients and factors related to readmissions in 2009. The results showed all-cause readmission rates were 13.6%, 23.6%, and 31.6% at 30-, 90- and 180-days respectively. The factors related readmissions included age, income, discharge/transfer status from index hospitalization, and comorbidity. Findings from this analysis suggested additional studies using multiple data sources are needed to have a comprehensive understanding of risk factors related HF patients’ healthcare utilization.

## Introduction

Heart failure (HF) is one of the most common chronic and disabling illnesses, resulting in high morbidity and mortality, caregiver burden and mental distress combined with challenges in disease management and access to appropriate healthcare services [1–3]. It is estimated that more than 8 million American will be living with HF by 2030 [4]. The total medical cost of care in 2030 will be $53.1 billion, and approximately 80% of the cost is related to hospital-based care [4].

HF is the leading cause of hospitalization in older adults aged 65 or greater in the United States [5]. Reducing HF patient readmission rates is an important national priority, as a low readmission rate is one of the primary indicators of health care quality and efficiency for HF patients [6,7]. Given the impact of HF on the healthcare system and patients’ quality of life, identifying factors that predict readmissions for HF patients is crucial in order to develop effective interventions and policies.

A literature review found inconsistent evidence for the role of specific factors that predict readmission of HF patients. For instance, O’Connor and Giamouzis found that patient characteristics had a small effect on readmissions [8,9], while others found that patient characteristics, such as age, gender, and comorbidity, were the strongest factors associated with readmissions [10]. The impact of rural residence on HF patients’ outcomes has been widely studied [11–14]. The findings are mixed. Several studies [12–15] reported higher readmission rates in rural HF patients, while others found patients living in rural communities had fewer readmissions [16]. The conflicting evidence could be attributed to the inherent limitations among existing studies in terms of cohort selection, length of follow-up, and data sources [8,17]. First, many studies reported the risk factors of readmissions in sub-populations of HF patients, such as Medicare/Medicaid beneficiaries [18–25], nursing home residents [26,27], veterans [28], and frail HF patients referred for hospice care [29,30]. Second, there are also variations in defining heart failure patients. Most studies included hospitalized patients with primary diagnoses of HF, leaving out those HF patients admitted for non-HF related admissions [31,32]. This is significant, since it has been suggested that patients admitted for HF as secondary diagnosis had higher mortality rate [33]. To capture a comprehensive cohort of HF patients at risk for adverse health outcomes, all diagnosis codes should be used to identify HF patients. Third, most studies focused on short-term impact of index hospitalization on subsequent readmissions (i.e., 30-day readmission) [8,18,34–37]. There is inadequate evidence on factors related to long-term impact of index admission on readmission. Finally, the risk factors of readmission based on investigator driven data often lack generalizability due to single-site, convenient, and small sample size [38,39]. Therefore, it was suggested using all-payer data that provide comprehensive views of risk factors related to readmissions [40]. Coffey et al. conducted a study to examine factors predicting readmissions in HF patients using multiple state inpatient data from the Healthcare Cost and Utilization Project (HCUP) [17], which is a nationally representative, all-payer database containing hospitalized patients’ demographic, clinical, and cost information over time [41]. However, Coffey’s study only included HF patients who were admitted to home healthcare agencies. HF patients referred to home health agencies are more likely to have poorer functioning, greater disease severity, and higher readmission rates [17]. In addition, Coffey’s study only included patients whose primary discharge diagnosis was HF [17], which could potentially overestimate HF patients’ readmission rates. Moreover, the study utilized the incomplete International Classification of Diseases-9 (ICD-9) codes to identify HF patients (i.e., nine ICD-9 codes, 402.01 through 404.93 were not included) [17].

Starting in 2010, the Affordable Care Act required Centers for Medicare & Medicaid Services (CMS) to reduce payments for readmissions in HF patients. To develop an effective readmission reduction program, it is critical to have full understanding of the risk factors for readmission in the HF population. Therefore, we examined the characteristics of hospitalized HF patients using Nebraska HCUP data, which provides a census database of all inpatient admissions in the state and, thus, permits a population-based perspective on HF patient care needs [17,19,20]. Our study is descriptive rather than predictive, by associating a large set of patient, clinical, and administrative factors to HF patient readmission outcomes. The effect of rural residence on healthcare utilization in HF patients has not been well understood, therefore, we chose HF patients residing in Nebraska because of large population of HF patients living in rural communities [42]. We chose 2009 data to examine healthcare utilization pattern because CMS began publicly reporting 30-day readmission rates for HF patients after 2009, which could potentially impact readmission rates [43]. This exploratory study helped to establish baseline data for future study of the impact of public reporting on readmission rate.

## New Contributions

Medicare policy that publicly reports and penalizes hospitals with high rates of readmissions [7] has been implemented since 2010. Examining the health care utilization pattern using 2009 data will serve as baseline data to assess the potential impact of policy changes on readmissions. Unlike others, our study had a more complete cohort of HF patients by including any hospitalized patients aged 19 years or above with HF as one of the discharge diagnoses. We used an all-payer database to provide comprehensive view of factors predicting readmission. Compared to other studies, we had longer follow-up of readmissions and identified different factors associated with both short and longer term readmissions.

To achieve this objective, we addressed the following research questions:

1. What is the pattern of discharge disposition from the index hospitalization?
2. What are the all-cause readmission rates at 30-, 90- and 180-days?
3. What were patient and index hospitalization characteristics associated with 30-, 90- and 180-day readmissions?

## Methods

### Study design

This is a retrospective cohort study of adults hospitalized and having heart failure as one of their discharge diagnoses for any hospitalization in the calendar year 2009.

### Data sources

The State Inpatient Database (SID) for Nebraska, part of the Healthcare Cost and Utilization Project (HCUP), was analyzed for this study. Use of the data was exempted from Institutional Review Board review by the UNMC Office of Regulatory Affairs. The SID includes all inpatient discharge records from hospitals in the state, regardless of payer, and provides a unique view of inpatient care, which can also inform post-acute care service planning for a defined population. HCUP is a Federal-State-Industry partnership sponsored by the Agency for Healthcare Research and Quality to inform decision making at the national, state, and community levels [44].

### Study population

**Figure 1:**
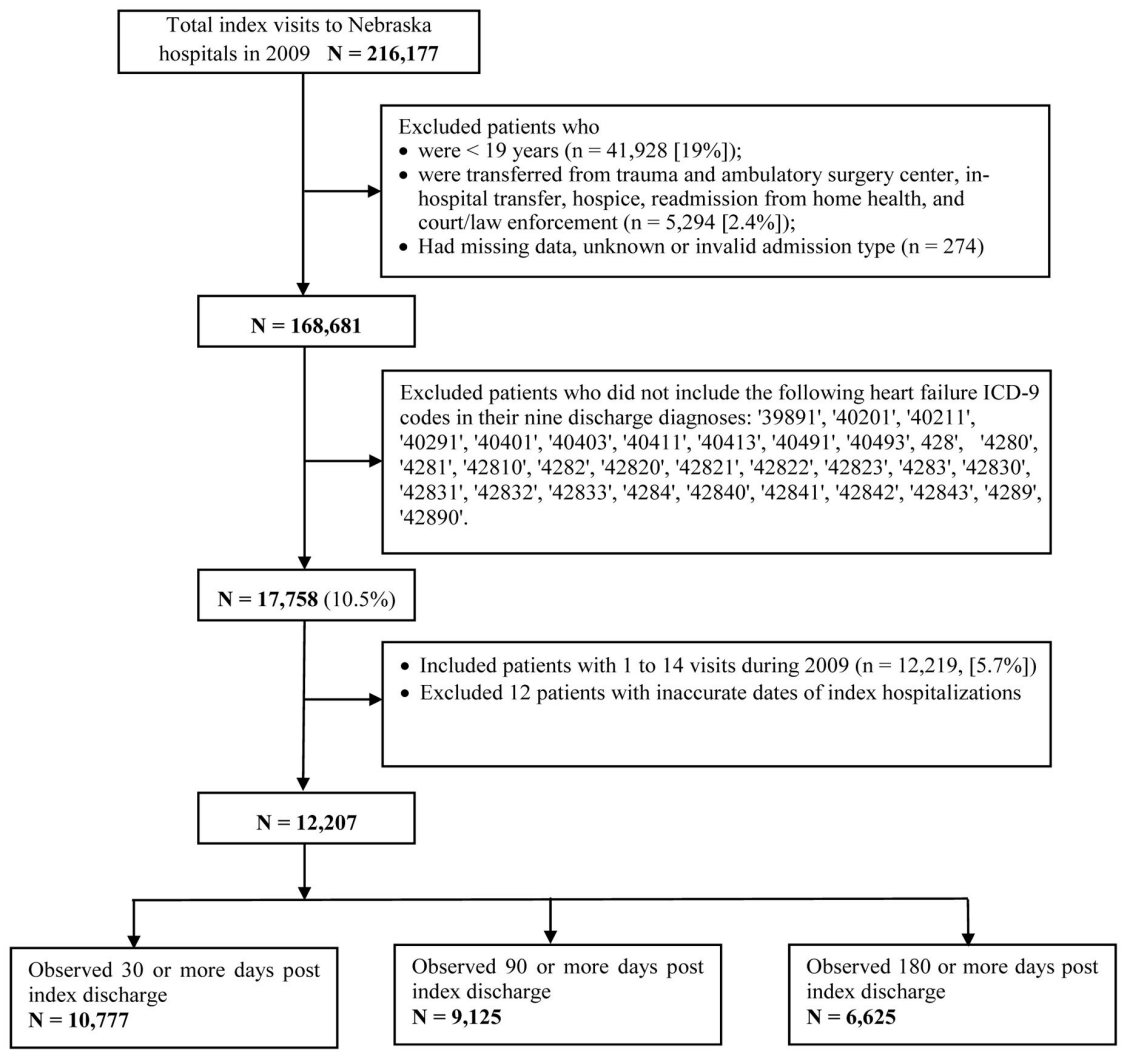
Overview of the selection of analysis cohort flowchart.

The population used for this study was selected from patients discharged from all Nebraska hospitals between January 1 and December 31, 2009 (n = 216,177 hospital discharges). Hospitalizations were excluded if 1) the patient was less than 19 years of age at discharge; 2) they were admitted from Trauma Centers, another acute setting, or an ambulatory surgery center; 3) the admission type was unknown or invalid; 4) their point of origin was unknown; 5) it was an in-hospital transfer; or 6) being readmitted from home health/hospice agencies or law enforcement (Figure 1). Of the remaining 168,681 hospitalizations, 17,785 (10.5%) had HF as at least one of the nine possible diagnosis codes ([ICD-9] codes: 398.91, 402.01, 402.11, 402.91, 404.01, 404.03, 404.11, 404.13, 404.91, 404.93, 428.0, 428.1, 428.20, 428.21, 428.22, 428.23, 428.30,428.31, 428.32, 428.33, 428.40, 428.41, 428.42, 428.43, and 428.9). These hospital visits were made by n = 12,219 individual patients, each with 1 to 14 visits during the year. The patient’s first hospitalization is referred to as the index visit. We excluded twelve patients whose second admission was recorded as occurring before their first discharge. A cohort of 12,207 heart failure patients comprised the analytical sample. In calculating 30-day readmission rates, patients were excluded if their index discharge occurred in December 2009, which was too late to determine 30-day post-discharge readmission, or if they died during the index admission. A total of 9,125 HF patients who were observed 90 days were used to compute 90-day readmission rate, while 6,625 patients observed for 180 days were the denominator for 180-day readmission rate. For logistic regression analyses, patients with missing information on any variables in the model were excluded (Figure 1).

### Variables

The primary outcome variables are 30-, 90-, and 180-day readmissions following the index hospitalization. Readmission is defined as the all-cause subsequent hospital stay following the index hospitalization [45]. Patient sociodemographic variables (i.e., age in years, gender, location of residence [urban vs. rural], household income quartile at zip code of patient residence) were included in the analysis. Based on the definition and classification method recommended by Rural Urban Commuting Areas (RUCA), a 4-level classification system is used in HCUP data to distinguish rural and urban residents. Due to small sample size, we combined Level 2–4 (large rural, small rural and isolated rural areas) into one category (i.e., rural), which has a range of RUCA values from 4 to 10.5. RUCA values are determined by the following criteria: 1) population size and rural ZIP Codes; 2) the frequency of commuting to larger urban areas; 3) the size of the urban destinations [46]. Clinical variables included length of stay in number of nights, whether or not the patient had a major operation or procedure during the index visit, comorbidities, and having HF as primary or secondary diagnosis. Discharge disposition or “discharge status” refers to the location where the patient is discharged after index hospitalization, including home, home with home health, and post-acute care settings (e.g., skilled care service in rural critical care hospitals or long term care facility, rehabilitation facility, swing bed program, etc.) [47]. Index hospitalization variables included total charges in dollars and primary payer (Medicaid, Medicare, and others, such as private payer, self-pay, and other federal and state health insurers).

### Data analysis

The HCUP dataset of discharge events was restricted according to the exclusion criteria listed above and restructured into a patient-level dataset based on codes representing patients and event dates (Figure 1). Descriptive statistics were calculated to describe HF population in terms of sociodemographic, clinical, and administrative characteristics such as charges, length of stay, and type of discharge (Table 1). The 30-, 90-, and 180-day readmission were defined as readmissions within 30-, 90-, and 180-day of index discharge respectively. To identify predictive factors of readmission, multivariable logistic regression analyses modeled 30, 90, and 180-day readmission. Independent variables were chosen based on literature review, including age, gender, location of residence (urban vs. rural), household income quartile at zip code of patient residence, primary payer, discharge disposition, comorbidities, whether heart failure was the first or second diagnosis on the index visit, and total charges of the index admission.

## Results

### Patient and index hospitalization characteristics

Characteristics of the cohort of 12,207 unique HF patients who had at least one hospitalization during 2009 were described. They were most likely to be between 65 and 85 years of age (55.4%) and Medicare beneficiaries (82.6%). Over half are female (51.5%). More than 90% HF patients lived in Nebraska (92.1%). A total of 54.4% of them resided in non-urban areas, such as large, small rural towns and isolated rural areas, while 58.7% of them lived in zip codes with median incomes below the median income for the US.

For the index hospitalization, approximately 90% patients were admitted from non-healthcare settings (i.e. home). The average length of stay was 5.33 (± 4.95) days, and the average hospital charge per patient was $36,212 (± $47,612). The in-hospital mortality rate was 4.7%. One in four (24.7%) had HF as their first (primary) diagnosis and more than 50% were admitted for non-HF related problems. Other common first diagnoses included pneumonia, atrial fibrillation, chronic obstructive pulmonary disease, myocardial infarction, coronary artery diseases, acute renal failure, and respiratory failure. One in five patients (20.3%) had a major operating room procedure during their index hospitalization. The most common comorbidity was hypertension (47.6%), followed by chronic pulmonary disorders (27.3%), diabetes (26.9%), fluid and electrolyte disorders (22.4%), and renal failure (19.8%) (Table 1).

#### Research question 1: The pattern of discharge disposition following the index hospitalization

**Figure 2:**
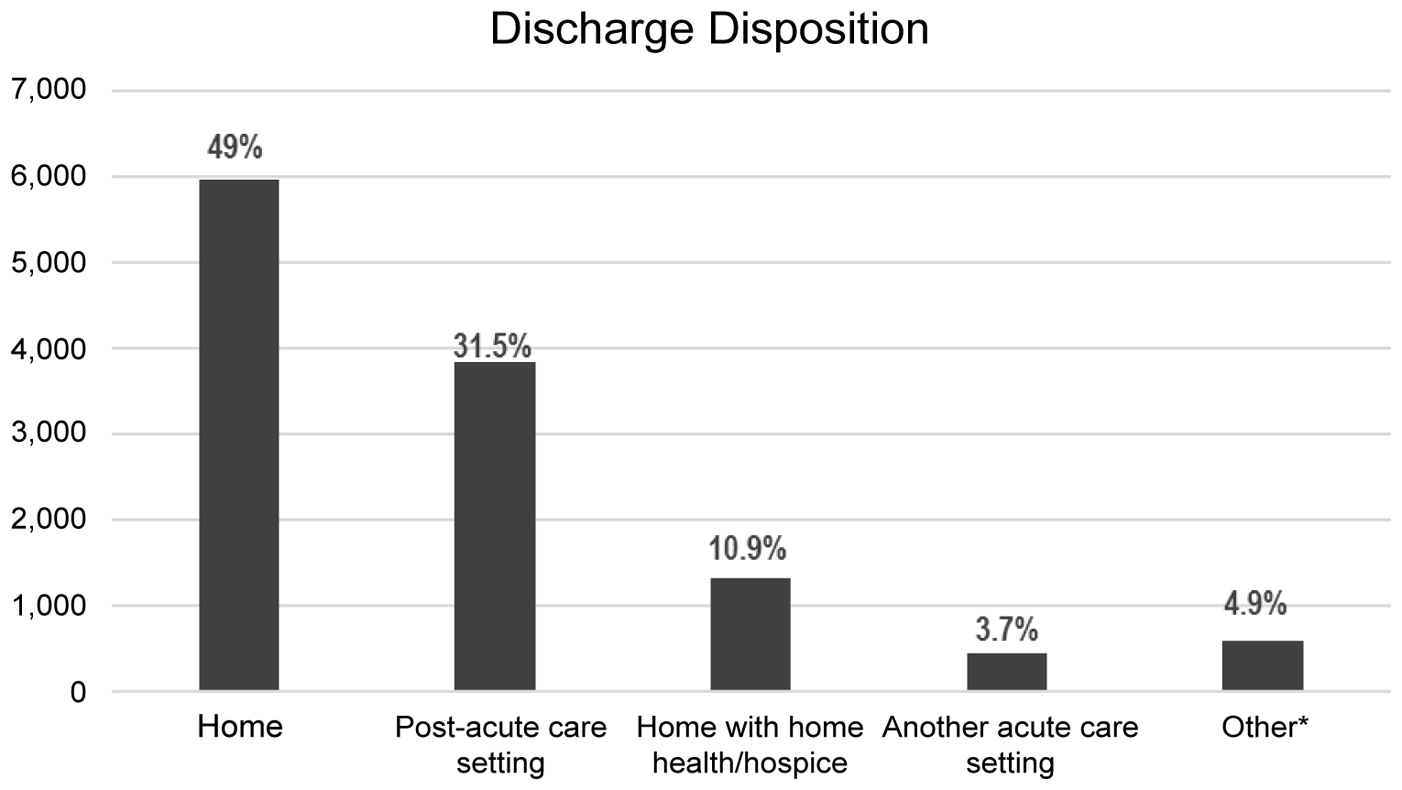
Discharge disposition for index hospitalizations (n = 12,207). ^*^Included the patients discharged with the status of against medical advice or to court/law enforcement or psychiatric facilities.

Among 12,207 HF patients, nearly half (5,975 [49%]) were discharged to home, more than 30% of heart failure patients (3,848 [31.5%]) were discharged to post-acute care facilities, and about one-tenth (1332 [10.91]) were discharged with home health agencies. Less than 5% (456 [3.7%]) of patients were transferred to another acute care hospital (Figure 2).

#### Research question 2: The all-cause readmission rates at 30-, 90- and 180-days

Of the 10,777 HF patients observed 30 or more days, 13.6% (n = 1,464) were readmitted within 30 days of index discharge. Among 9,125 HF patients observed 90 or more days, a total of 2,158 (23.6%) were readmitted within 90 days of discharge, and 31.6% (n = 2109) patients observed more than 180 days (n = 6,625) were readmitted within 180 days.

#### Research question 3: patient and index hospitalization characteristics associated with 30, 60, and 90 day readmissions

Multivariable logistic regression estimated the effects of patient and index hospitalization characteristics on 30-, 90-, and 180-day readmissions, adjusted for all other factors in the model (Table 2). Based on 2009 data, the higher odds of readmission within 30-days of index discharge for HF patients occurred among the oldest age group (e.g., being 86 years of age or older) compared to younger age group (odds ratio [OR], 1.313; 95% CI, 1.04–1.65), and among those being transferred to another acute care setting (OR, 6.507; 95% CI, 5.21–8.13), being discharged to post-acute care setting (OR, 1.307; 95% CI, 1.13–1.51) or home health setting (OR, 1.364, 95% CI, 1.13–1.64). The HF patients who had total charges of index admission more than $40,001 had increased odds of 30-day readmission (OR, 1.329; 95% CI, 1.10–1.61) compared to those who spent less ($776 – $10,000) for index visit. On the other hand, having major operation/procedure done at index visit decreased the odds of 30-day readmission (OR, 0.605; 95% CI, 0.51–0.72).

The HF-related index admission increased both odds of 30-day (OR, 1.345; 95% CI, 1.20–1.51) and 90-day (OR, 1.220; 95% CI, 1.06–1.41) readmissions. Being transferred to another acute care hospital increased odds of being readmitted within 30 days (OR, 6.507; 95% CI, 5.21–8.13), but reduced odds of 90-day (OR, 0.338; 95% CI, 0.19–0.60) and 180-day (OR, 0.283; 95% CI, 0.13–0.61). Similarly, being discharged to post-acute care setting increased odds of 30-day readmission (OR, 1.307; 95% CI 1.13–0.51), but reduced odds of 180-day (OR, 0.759; 95% CI, 0.60–0.96) readmissions.

The comorbidities that were associated with increased odds of 30-day readmission include fluid and electrolyte disorder (OR, 1.176; 95% CI, 1.03–1.35) and renal failure (OR, 1.398; 95% CI, 1.22–1.60). Comorbidities increased odds of 90-day readmission include hypertension (OR, 0.842; 95% CI, 0.73–0.97) and renal failure (OR, 1.269; 95% CI, 1.07–1.50). Having comorbidities of blood disorder (OR, 2.838; 95% CI, 1.44–5.58) or obesity (OR, 1.484; 95% CI, 1.08–2.05) increased the odds of being readmitted within 180 days.

### Sensitivity analysis

We used different HF cohorts for each logistic regression model for 30 day (n = 10,672), 90 day (n = 9,038), and 180 day (n = 6,562) readmissions. Because different cohorts were associated with each 30, 90, and 180 day outcome variable, it would not be accurate to directly compare findings across models. To account for this variation in samples for each model, we performed a sensitivity analysis using the same HF cohort for all three models (n = 6,625). There was no major differences between the logistic regression models.

## Discussion

Unlike other studies using Medicare claims data [19] or identifying HF cohorts based on the primary diagnosis of index admission [48], we identified the HF population by including all patients who were 19 years or older who had HF as one of the documented discharge diagnoses. In addition, the all-payer data were used to capture a comprehensive view of healthcare utilization characteristics in HF patients. Our study showed almost 50% of HF patients were discharged from the index hospitals to other healthcare facilities (e.g., post-acute care settings, another acute care hospitals) or home health agencies. The risk factors associated with 30-day readmissions included older age, being discharged to home with home health agencies, HF-related index admission, and having complications or multiple comorbidities (i.e., fluid and electrolyte disorders and renal failure). HF-related index admission and comorbidity of renal failure increased risk of 90-day readmission, while comorbidity of blood disorder and obesity was associated with 180-day readmission.

Due to wide range variations in cohort selection, data source, and readmission reporting, it is difficult to compare our readmission rate with other studies. The majority of studies focused on 30-day readmission with a median rate of 21% (range = 2%–52%) [8]. Few studies within the past 10 years reported 90- and 180-day readmission rates [8].

O’Connor et al. [8] conducted a comprehensive literature review and found there was no consistency in the effect of patient demographic factors on readmission. Except for age, there was no significant association between readmission rates and other sociodemographic factors in our study (e.g., zip code median income, gender). For factors related to index hospital stay, our data showed a similar proportion of non-home discharge (more than 50%) in HF patients [22]. This may reflect HF patients’ increased needs for additional post-acute care services immediately following hospital discharge because of the Medicare’s Prospective Payment System policy to incentivize shortened length of stay [8,49,50].

Disease severity and acuity at index admission have been widely accepted as one of key risk factors of 30-day readmission [8,17]. The proxy measures of disease severity and acuity in our analysis were discharge/transfer disposition and the total charge of index stay [17,51–53]. Similar to Madigan’s findings [10], we found that HF patients discharged to home health series had greater risk (36%) of 30-day readmissions, indicating their needs of hospital services within 30 days, but not 90- and 180-days. We also observed paradoxical relationships between post-acute care service use, between-hospital transfer and readmission. Post-acute care service use and between-hospital transfer following index visits increased risk of short-term readmission (30-day), but reduced the risk of long-term readmissions (90- and 180-day). The explanation may be that patients transferred to post-acute care or another hospital were more likely to have greater disease severity and complications, resulting in greater risk of adverse events soon after discharge (e.g., death). Another variable reflecting the disease severity is the cost of index admission. It was reported that the total hospital charges were significantly higher for patients with greater disease severity [17,54]. Similar to others’ findings [8,17] the total charges of index hospitalization were significantly related to 30-day readmission.

Like other studies reported [8,50,55], we also found multicomorbidity increased the likelihood of readmission at all time points. As reported by other studies [19,50,55,56], most HF patients have multiple chronic, complex comorbidities that create great challenges in discharge management. In addition, similar to others’ reports [34,37,57,58], more than 50% of index hospitalizations were not HF-related. Current standardized HF-specific treatment and discharge instruction guidelines are less likely to reduce all-cause hospitalizations in this population [59,60].

Unexpectedly, we observed a negative relationship between having major operations or procedures at index hospital stays and all readmissions (30-, 90-, and 180-days), which has not been reported in other studies. The explanations would be that patients undergoing major procedures during index admission were likely to be healthier; therefore, they were less likely to be readmitted within 30-days.

There are a number of limitations to the study. First, the study has little evidence about the healthcare utilization characteristics in HF patients from racially and ethnically minority groups, due to confounding effects with other variables, which affects the generalization of the results. Secondly, the major gap in knowledge about HF patient readmission risk factors is primarily the limited access to a more comprehensive profile of risk factors, such as individual-, provider- and hospital-levels of variables [8]. The use of an all-payer dataset alone does not sufficiently include all the variables associated with healthcare utilizations. For instance, lifestyle behaviors prior to the index hospital stay and social factors have been found the strong indicators of healthcare use [12,61]. However, these variables are often missing or not readily accessible in all-payer data, which creates a major gap in understanding the overall readmission risk factors in the HF population. Hospital level data (e.g., hospital size, ownership or dedicated cardiovascular center) and long-term care transitional care data were not available in this study. As a result, hospital-level quality of care measures, an important indicator of readmissions, were not controlled. Third, the lack of longitudinal data of healthcare utilizations in both outpatient and inpatient settings limits our understanding of how identified risk factors affect readmissions overtime. Fourth, The HCUP system allows for charges to be estimated based on a known charge-to-cost ratio for each hospital. That could not be done in this study, as each hospital was not identified in the dataset. Therefore, we used charge information, which is useful for comparison within this study, but is not comparable to cost information published in other studies based on HCUP data. Fifth, the model validity of the multivariate logistic regression was not optimal (see fit statistics in the Table 2). However, our findings are still noteworthy to report because the goal of the study is not to fit the best model, but to describe relationships among variables. Last, this is an observational study that is unable to establish causal links between associated factors.

### Recommendations for future research direction

Patient centered care calls for evidence on the multi-level and multifaceted factors associated with readmission, which helps develop effective strategies to reduce care system overload, cost, and improve care quality. Therefore, multiple data sources should be used, such as datasets including patient reported variables (e.g., functioning status, social support, health belief/behavior, etc), clinical and administrative variables. In addition, examining factors related to high frequency of readmissions is critical. Last but not least, post index discharge healthcare utilizations (e.g., home health and post-acute care services) are closely related to readmissions. Examining the pattern and indicators of post discharge healthcare utilization plays a vital role in identifying effective strategies to reduce readmission and improve care continuity in this population with multiple, chronic, and complex conditions.

## Conclusion

Our findings emphasize the challenges of identifying reliable and valid risk factors of readmission for HF patients. To develop effective readmission reduction interventions, additional studies are needed to examine the effects of both medical and social factors on HF patients’ healthcare utilization patterns. To have a comprehensive understanding of factors related to HF patients’ readmission, multiple data resources (e.g., health records, payers’ data, national health behavior survey data, and HF patient registries) should be utilized. This is a critical first step to improving quality of life for HF patients and attenuating healthcare expenditures as the prevalence of HF patients drastically increases.

## Figures and Tables

**Table 1:** Patient demographic, clinical, and administrative characteristics at index hospitalization (n = 12,207).

Variables	N (%) or Mean ± SD
**Demographic Variables**	
Age	75.93 ± 13.23
Age 19–64	2,339 (19.2)
Age 65–85	6,758 (55.4)
Age 86 and older	3,110 (25.5)
Female	6,281 (51.5)
Urban residents	5,457 (44.7)
Median household income national quartile	
First Quartile	2,891 (23.7)
Second Quartile	6,183 (50.7)
Third Quartile	2,109 (17.3)
Fourth Quartile	919 (7.5)
Primary payer on index visit	
Medicare	10,082 (82.6)
Medicaid	359 (2.9)
Other*	1,766 (14.5)
**Clinical Variables**	
Length of stay for index hospitalization	5.33 ± 4.95
Total length of stay in 2009	7.81 ± 8.26
Had major operation procedure on index visit	2,479 (20.3)
Mortality rate during index hospitalization	570 (4.7%)
**Selected Comorbidities**	
Hypertension	5,809 (47.6)
Chronic pulmonary disorders	3,331 (27.3)
Diabetes	3,286 (26.9)
Fluid and electrolyte disorders	2,732 (22.4)
Renal failure	2,412 (19.8)
Blood disorders	2,054 (16.8)
Hypothyroidism	1,099 (9.0)
Obesity	830 (6.8)
Other neurological disorders	718 (5.9)
Valvular disease	623 (5.1)
Peripheral vascular disorders	614 (5.0)
Depression	467 (3.8)
First diagnosis of heart failure	3,020 (24.7)
First or second diagnosis of heart failure	5,682 (46.6)
**Administrative Variables**	
Total charges of index hospitalization	36,211.82 ± 47,612.04
$776 to $10,000	2,593 (21.2)
$10,001 to $20,000	3,286 (26.9)
$20,001 to $40,000	3,082 (25.3)
$40,001 to $769,063	3,246 (26.6)
Total charges of all hospitalizations in 2009	50,877.31 ± 64,162.06
Admissions from from non-healthcare settings (e.g., home)	10884 (89.16)

* Other included private payer, self-pay, and other federal and state health insurers.

**Table 2:** Logistic model adjusted-odds ratios: association between readmissions within 30 days, 90 days, and 180 days of index admission for patients diagnosed with heart failure and patient demographic, clinical, and administrative characteristics.

Variables	Odds Ratios for Readmission^c^					
	30 Days (n = 10,672)		90 Days (n = 9,038)		180 Days (n = 6,562)	
	OR	CI	OR	CI	OR	CI
**Demographic Characteristics**						
Age (Reference = Age 19–64)						
Age 65–85	1.217	1.00 – 1.49	1.166	0.92 – 1.49	1.106	0.81 – 1.51
Age 86-high	1.313*	1.04 – 1.65	1.062	0.80 – 1.41	1.014	0.71 – 1.46
Female (Reference = Male)	0.929	0.83 – 1.05	0.879	0.76 – 1.02	1.069	0.89 – 1.29
Urban residents (Reference = rural residents)	1.103	0.96 – 1.27	1.235*	1.04 – 1.47	1.146	0.91 – 1.44
Median household income (Reference = First quartile)^a^						
Second Quartile	1.236**	1.07 – 1.43	1.078	0.90 – 1.29	0.906	0.72 – 1.13
Third Quartile	1.125	0.93 – 1.37	0.91	0.72 – 1.15	0.840	0.62 – 1.14
Fourth Quartile	1.144	0.89 – 1.48	0.867	0.63 – 1.19	0.960	0.64 – 1.43
Payer (Reference = Other)						
Medicare	1.135	0.92 – 1.40	1.120	0.87 – 1.45	1.341	0.95 – 1.89
Medicaid	1.228	0.85 – 1.78	1.128	0.70 – 1.80	1.788*	1.04 – 3.06
**Clinical Characteristics**						
Had major operation procedure on index visit (Reference = had no operational procedure on index visit)	0.605***	0.51 – 0.72	**0.948**	0.77 – 1.16	0.721*	0.54 – 0.96
Disposition/Transfer (Reference = Discharge to home)						
Transferred to another acute care setting	6.507***	5.21 – 8.13	0.338***	0.19 – 0.60	0.283**	0.13 – 0.61
Discharge to post-acute care setting	1.307***	1.13 – 1.51	0.882	0.74 – 1.05	0.759*	0.60 – 0.96
Discharge to home with home health/hospice	1.364**	1.13 – 1.64	1.149	0.92 – 1.43	1.102	0.83 – 1.46
Other^b^	3.457*	1.23 – 9.68	0.784	0.10 – 6.09	N/A	N/A
Selected Comorbidities (Reference = Absence of specific comorbidity)						
Hypertension	0.975	0.87 – 1.10	0.842*	0.73 – 0.97	0.917	0.76 – 1.11
Chronic pulmonary disorders	1.223	0.93 – 1.60	0.993	0.69 – 1.43	1.037	0.66 – 1.64
Diabetes	1.024	0.89 – 1.18	1.029	0.87 – 1.22	1.197	0.97 – 1.47
Fluid and electrolyte disorders	1.176*	1.03 – 1.35	1.103	0.93 – 1.31	0.82	0.65 – 1.04
Renal failure	1.398***	1.22 – 1.60	1.269**	1.07 – 1.50	1.149	0.92 – 1.44
Blood disorders	0.990	0.56 – 1.74	0.330	0.10 – 1.05	2.838**	1.44 – 5.58
Hypothyroidism	0.921	0.75 – 1.13	1.170	0.93 – 1.48	1.095	0.80 – 1.49
Obesity	0.885	0.69 – 1.13	1.099	0.84 – 1.45	1.484*	1.08 – 2.05
Other neurological disorders	0.840	0.65 – 1.09	0.991	0.73 – 1.35	0.976	0.65 – 1.47
Valvular disease	1.040	0.81 – 1.35	1.199	0.89 – 1.62	1.039	0.70 – 1.55
Peripheral vascular disorders	1.080	0.84 – 1.39	0.947	0.69 – 1.30	1.017	0.69 – 1.51
Depression	1.222	0.92 – 1.63	0.981	0.68 – 1.42	0.629	0.36 – 1.09
First or second diagnosis of HF on index visit (Reference = First or second diagnosis not related to HF)	1.345***	1.20 – 1.51	1.220	1.06 – 1.41	1.103	0.91 – 1.33
Administration Characteristics						
Total Charges of index hospitalization (Reference = $776 to $10,000)						
$10,001 to $20,000	0.859	0.72 – 1.02	1.079	0.88 – 1.32	0.922	0.71 – 1.19
$20,001 to $40,000	1.119	0.94 – 1.34	0.964	0.77 – 1.20	0.861	0.65 – 1.14
$40,001 to $769,063	1.329**	1.10 – 1.61	0.774*	0.60 – 0.99	0.772	0.56 – 1.06

a Median income of households in patient’s zip code (National Quartile, Reference=Lowest Income, first quartile).

b Other discharges included the patients discharged with the status of against medical advice or to court/law enforcement or psychiatric facilities.

c Model fit indices: R^2^ = 0.0644; Hosmer-Lemeshow Test (p = 0.0197); C-statistics = 0.6483 (95% CI 0.6325 – 0.6641)

* p < 0.05,

** p < 0.01,

*** p < 0.001
